# Supplementary material for: Molecular dynamics study on the effects of charged amino acid distribution under low pH condition to the unfolding of hen egg white lysozyme and formation of beta strands
Source: PLoS One. 2022 Mar 24;17(3):e0249742. doi: 10.1371/journal.pone.0249742 (PMC8946743; doi:10.1371/journal.pone.0249742)
Supplement: S1 File — (DOCX) [file pone.0249742.s001.docx]

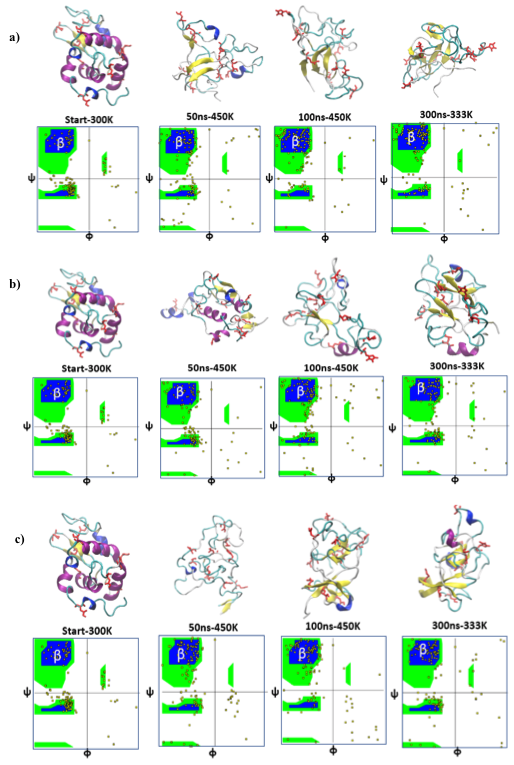


**S1 Fig.** Conformational snapshots of HEWL at pH2 (top) and ramachandran plots (bottom) for the replicas (a) R0, (b) R1, and (c) R2 taken from at the start, after 50 ns of simulations at 450 K, after 100 ns of simulations at 450 K, and after 300 ns of simulations at 333 K.


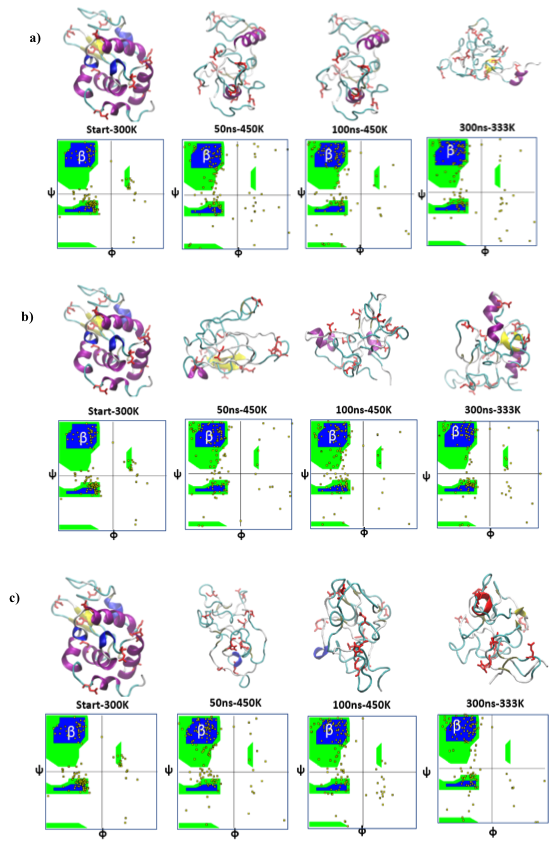


**S2 Fig.** Conformational snapshots of HEWL at pH7 (top) and ramachandran plots (bottom) for the replicas (a) R0, (b) R1, and (c) R2 taken from at the start, after 50 ns of simulations at 450 K, after 100 ns of simulations at 450 K, and after 300 ns of simulations at 333 K.
